# *AI-Enabled Sacramento Public Health (SACPH) App:* A Reproducible AI-Based Method for Population-to-Practice Reasoning in Foundational Sciences in Pharmacy Education

**DOI:** 10.3390/pharmacy14010010

**Published:** 2026-01-16

**Authors:** Ashim Malhotra

**Affiliations:** Department of Pharmaceutical and Biomedical Sciences, California Northstate University College of Pharmacy, 9700 W Taron Drive, Elk Grove, CA 95758, USA; ashim.malhotra@cnsu.edu; Tel.: +1-916-686-8885

**Keywords:** immunology, pharmacy education, epidemiology, generative AI app, educational app

## Abstract

Foundational biomedical sciences are commonly taught without routine integration of local population health contexts, limiting students’ ability to connect mechanisms to community disease burden and practice responsibilities. In this method paper, we developed and piloted an AI-enabled “Sacramento County Public Health (SACPH)” AI workflow and app prototype, a structured, faculty-authored prompt sequence designed to guide population-to-practice reasoning using publicly available data. The workflow was implemented during a TBL session with first-year PharmD students in an immunology course. Using splenectomy and risk of overwhelming post-splenectomy infection (OPSI) as an illustrative use case, students executed a standardized prompt sequence addressing data source identification, coding logic (diagnosis vs. procedure codes), population-level estimation with uncertainty framing, and translation to pharmacist-relevant prevention and counseling implications. Feasibility was defined by conceptual convergence. The validated reasoning workflow was subsequently translated into a prototype, app-style interface using generative design prompts. Across student teams, outputs converged on similar categories, consistent recognition of coding frameworks and verification steps, and directionally similar interpretations of local burden and pharmacist responsibilities. The prototype demonstrated successful externalization of the reasoning workflow into a modular, reproducible artifact. SACPH demonstrates a feasible, reproducible method for using generative AI to integrate foundational science instruction with local population health context and pharmacist practice reasoning, while supporting AI literacy competencies.

## 1. Introduction

Health professions education has long emphasized mastery of foundational biomedical sciences as a prerequisite for safe and effective clinical practice [1]. However, these foundational concepts are frequently taught in isolation from the epidemiologic realities of the communities in which learners will ultimately practice [2]. As a result, students often acquire detailed mechanistic knowledge of disease without an accompanying understanding of how frequently relevant conditions occur locally, which patient populations are most affected, or how professional responsibilities intersect with community-level health needs. This disconnect is particularly consequential in pharmacy education, where practitioners are expected to anticipate population risks [3], engage in prevention [4], and provide community-responsive counseling, yet are rarely trained to reason explicitly from local disease burden to practice priorities. The need for pharmacy graduates to be able to gauge, assess, and deliver population-level health is enshrined in the 2025 ACPE accreditation guidelines [5].

Immunology provides a particularly instructive example of this challenge. As a foundational discipline, immunology is conceptually abstract, systems-oriented, and mechanistically dense [6], requiring learners to integrate molecular pathways, cellular interactions, and host responses that can feel clinically distant at the time of instruction. Consequently, immunology is frequently experienced by learners as difficult to apply when taught without explicit linkage to practice relevance, frequency of encounter, or community disease burden [6,7]. However, immunologic mechanisms underpin common clinical conditions and preventive interventions that pharmacists address routinely. From the perspective of adult learning theory [8], the absence of contextual “why” weakens engagement and limits the perceived value of foundational content.

Despite widespread calls to strengthen population health education, local, practice-relevant epidemiologic data are rarely integrated into foundational science teaching in a routine and scalable way [9]. As pharmacists increasingly train and practice within multistakeholder teams addressing population-level health challenges, foundational biomedical sciences must be taught in ways that extend beyond isolated clinical encounters to support interpretation of public health data and participation in collaborative care. Collaborative care requires pharmacists to discuss topics relevant to population care, medication management, and social, environmental, and economic determinants of health [10]. Despite this, population health is often siloed into standalone courses, while foundational disciplines rely on national-level examples, textbook descriptions, or decontextualized mechanisms [1]. This dichotomy creates learner cognitive dissonance. Furthermore, a practical barrier to the seamless integration of foundational sciences with public health and public health data is that faculty and learners frequently lack time and technical expertise to locate relevant public datasets, interpret coding frameworks, and translate administrative or surveillance data into practice-oriented insight [11].

Recent advances in generative artificial intelligence (AI) provide an opportunity to remove this barrier. Large language models can help identify public data sources, explain how administrative data are structured, scaffold interpretation, and translate estimates into narrative practice implications—provided the interaction is disciplined and constrained. Importantly, AI does not create a new educational goal; it enables a long-standing goal that has been impractical at scale: connecting what we teach to what is prevalent where students live and will practice.

This study describes the development and pilot feasibility of AI-enabled SACPH (Sacramento County Public Health), a prompt-based AI workflow designed to integrate foundational science content with local population health context and pharmacist-relevant reasoning. The SACPH workflow was conceived in response to this gap, using immunology as an initial test case to explore whether AI-assisted access to local epidemiologic context could make foundational science instruction immediately meaningful by explicitly linking mechanisms to community disease burden and pharmacist responsibility. The workflow was grounded in adult learning principles and aligned with Miller’s Pyramid [12], supporting progression from “knows” to “knows how/shows how” through structured inquiry rather than outcome testing. Using splenectomy and post-splenectomy infectious risk as an illustrative use case, the study further demonstrates how the same AI-guided reasoning structure can be translated into a prototype interface specification intended to support broader dissemination.

## 2. Methods

### 2.1. Study Design and Guiding Frameworks of Adult Learning and Miller’s Pyramid

This project employed a methods-focused feasibility design to develop, pilot, and document an AI-assisted instructional workflow that connects foundational biomedical science instruction to local population health context and pharmacy practice implications. The intent was not to evaluate learner outcomes, but to demonstrate the feasibility and reproducibility of a structured AI-guided inquiry process when executed by faculty and students using standardized prompts.

Instructional rationale was grounded in adult learning theory (purpose-driven, problem-centered learning) [8] and aligned with Miller’s Pyramid [12] of clinical competence, supporting progression from foundational knowledge (“knows”) toward applied reasoning (“knows how/shows how”) without claiming independent clinical performance (“does”).

The SACPH workflow was operationalized to embed specific tenets of adult learning theory and Miller’s Pyramid while simultaneously introducing foundational AI literacy and prompt-engineering skills.

Adult learning principles of relevance and self-directed inquiry were enacted by requiring learners to initiate analysis with community-specific disease burden and to interrogate public data sources using structured prompts rather than receiving curated answers. As shown in Table 1, alignment with Miller’s Pyramid was implemented through deliberate task sequencing that also maps to emerging AI competencies: identification of official data sources and foundational concepts (“knows”) corresponds to understanding what questions can appropriately be posed to AI systems; interpretation of coding frameworks and population-level estimates (“knows how”) requires learners to refine prompts, assess AI justification, and recognize uncertainty; and translation of epidemiologic context into pharmacist-relevant preventive and counseling implications (“shows how”) requires learners to use AI outputs critically in applied professional reasoning. In this way, SACPH integrates traditional models of clinical competence with practical AI literacy by embedding prompt construction, verification, and interpretation directly into the instructional method.

### 2.2. Setting and Instructional Context

The workflow was implemented during a team-based learning (TBL) session in a Doctor of Pharmacy curriculum delivered in a three-year PharmD program. The activity was conducted with first-year (P1) PharmD students within an immunology course. Students worked in teams to ensure equitable access to AI tools.

The clinical anchor was splenectomy and the risk of overwhelming post-splenectomy infection (OPSI), selected because it links immunologic mechanisms (splenic immune function), population-level procedure prevalence, clinically meaningful risk estimates, and pharmacist responsibilities related to vaccination counseling and prevention [13,14,15]. Please note that OPSI is simply relevant in this methods paper as it is the clinical manifestation of what may occur in patients after splenectomy, and pharmacists must counsel for vaccination. Instead of OPSI, any other clinical follow-up may be used based on the specific disease.

#### AI Platform and Operational Constraints

All interactions were conducted using ChatGPT4.0 (OpenAI, San Francisco, CA, USA) via the standard browser interface. No API access, automation, programming, or external tooling was used. The workflow was restricted to publicly available information and educational purposes only. Students were instructed that AI outputs were to be treated as a scaffold for inquiry and verification, rather than as an authoritative source for clinical decisions.

### 2.3. The Feasibility Instrument: SACPH Prompt Sequence Used by Faculty and Students

The methodological unit of this study was an AI-guided inquiry instrument: a fixed sequence of prompts authored by the faculty investigator and distributed to students via the learning management system. Students were instructed to copy/paste prompts verbatim.

The prompt sequence was designed to scaffold four functions: (i) identification of official data sources, (ii) translation of clinical concepts into coding logic used in public/administrative datasets, (iii) estimation and contextualization of prevalence and risk, and (iv) translation into pharmacist-relevant implications.

**SACPH AI-guided inquiry instrument (core prompts)**

*(Prompts are shown in publishable form; local parameters are bracketed for replacement.)*

A *Data source discovery and verification*
  1. “List official public datasets or dashboards that could support estimating the number of splenectomies performed in [Sacramento County] for [2020–2023]. For each source, explain why it is official and how to confirm provenance (agency owner, update cadence, methodology notes).”
  2. “What are the common pitfalls in using public health dashboards for procedure counts? Provide a short checklist for verifying that a source is official and that a measure represents procedures performed rather than diagnoses recorded.”
B *Coding logic (why codes matter)*
  3. “Explain how splenectomy would be represented in administrative datasets. Distinguish diagnosis codes vs. procedure codes (e.g., ICD-10-CM vs. ICD-10-PCS, and CPT). What does each coding system capture, and what are the limitations?”
  4. “Provide candidate procedure-code concepts relevant to splenectomy and explain how a user should verify the exact code set used in a given dataset. Do not invent a single ‘correct’ code—explain the verification process.”
C *Estimation and interpretation*
  5. “If a dataset provides counts by year, show how to compute the total number of splenectomies in [Sacramento County] across [2020–2023], and explain uncertainty sources (coding changes, reporting boundaries, missing data).”
  6. “OPSI (overwhelming post-splenectomy infection) is often described as a low-frequency but high-severity risk. Using published benchmark ranges (without claiming patient-specific prediction), explain how one could translate a county-level splenectomy count into a rough estimate of expected OPSI burden over time, clearly stating assumptions.”
D *Translation to pharmacist practice*
  7. “Translate the epidemiologic estimate into pharmacist-relevant implications: which practice settings are most likely to encounter post-splenectomy patients, what preventive care actions are relevant (e.g., vaccine counseling), and what a pharmacist should anticipate in local practice.”
  8. “Generate a brief ‘community-facing’ explanation (2–3 sentences) that a pharmacist could use to explain why post-splenectomy prevention matters, written for a general audience.”

Faculty reference execution and refinement.

Prior to classroom implementation, the faculty investigator executed the full prompt sequence to establish a reference pattern of responses and refine prompts for disciplined behavior. Prompts were revised to force provenance checks, discourage false numerical precision, and elicit verification steps rather than confident but unsupported claims.

### 2.4. Classroom Feasibility Test and Operational Definition of Reproducibility

During the TBL session, student teams executed the standardized prompt sequence and recorded outputs. Feasibility was defined as the ability of the inquiry instrument to produce coherent, practice-relevant reasoning using public information within the time constraints of a classroom session.

Reproducibility was defined as convergence in (i) types of official sources identified, (ii) recognition of coding frameworks and verification steps, (iii) directionally similar prevalence/risk interpretations, and (iv) consistent linkage to pharmacist responsibilities—without requiring identical wording across teams.

### 2.5. Safeguards Addressing AI Limitations

Safeguards included faculty-curated prompts, explicit constraints to public sources, prompts that required verification language, and in-class faculty oversight to identify and correct unsupported claims. The workflow was designed to model disciplined data literacy behaviors: verifying provenance, acknowledging uncertainty, and distinguishing estimates from validated counts.

### 2.6. Translating Workflow into a Prototype Interface Specification

To demonstrate scalability beyond text-based interaction, the validated reasoning structure was translated into a prototype interface specification using a structured generative design prompt. The goal was not to produce operational software, but to externalize the workflow as an interface-level artifact that mirrors the inquiry logic.

**Figure 1:**
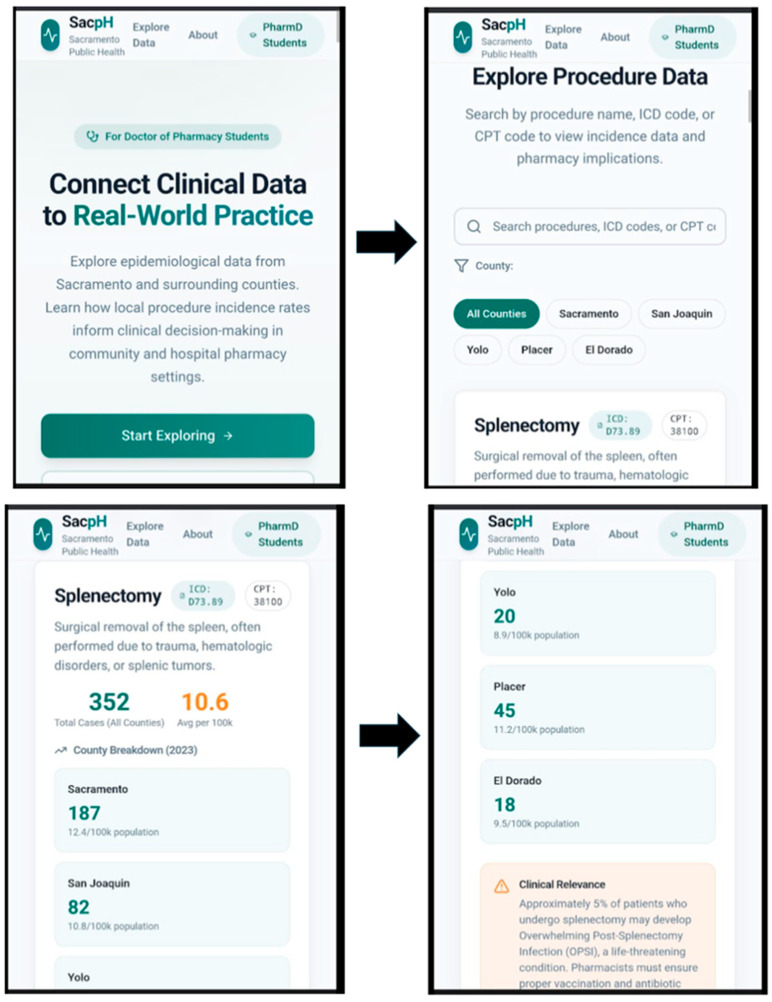
AI-generated prototype interface for population-to-practice reasoning (overview screen). Representative overview screen from the AI-generated prototype illustrating how local epidemiologic data, clinical context, and pharmacist responsibilities can be visually integrated into a single interface. The design emphasizes geographic specificity, procedure frequency, and downstream clinical implications, supporting learner reasoning from foundational immunologic concepts to population-aware pharmacy practice.

**Figure 2:**
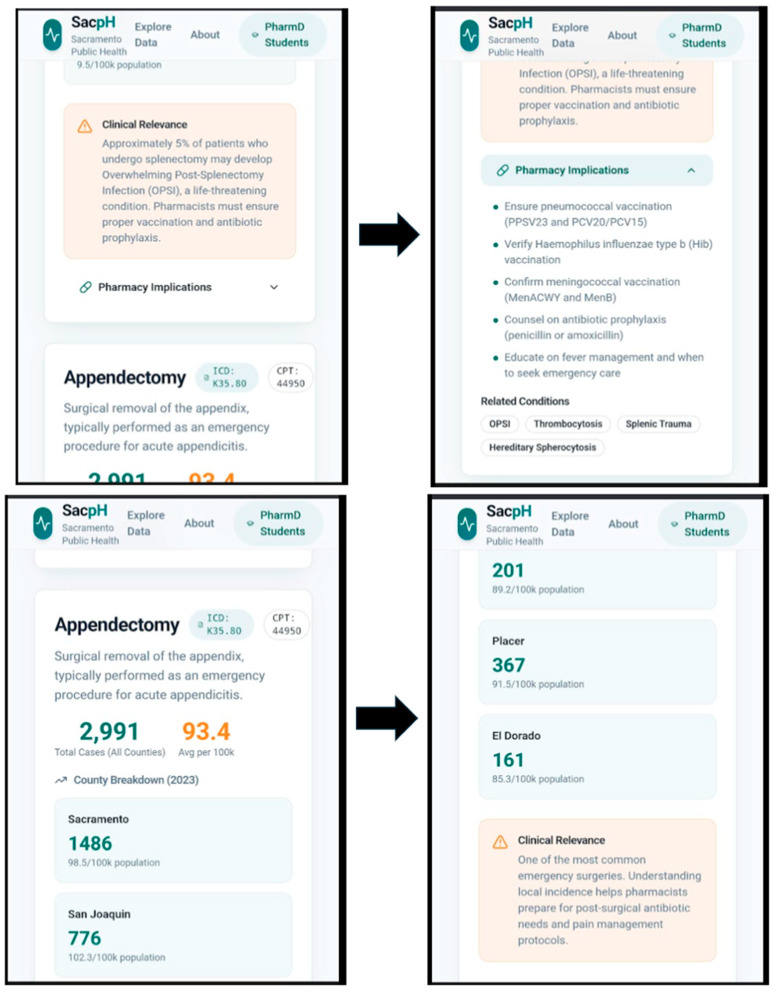
Extension of the SACPH workflow to an alternative clinical condition (appendectomy). Screenshots taken on a cellphone illustrating the generalizability of the SACPH design framework beyond immunology surfaced through a demo SACPH app. The appendectomy example demonstrates reuse of the same AI-assisted workflow to identify procedure prevalence, population burden, and downstream pharmacist-relevant considerations, supporting scalability of the approach across disease states and foundational science domains. The screenshots show how the app appears as a user scrolls down, thus the truncated text and images are intentionally depicted.

Screenshots of the resulting prototype are presented in the Results (Figure 1 and Figure 2) as evidence of translation feasibility.

## 3. Results

### 3.1. Feasibility of the AI-Guided Inquiry Instrument

The standardized SACPH prompt sequence successfully guided the AI system to generate structured outputs spanning data source identification, coding logic explanation, estimation framing, clinical risk contextualization, and pharmacist-relevant interpretation. Outputs routinely included provenance cues (who owns the dataset, how to verify official status) and produced narrative estimates framed with assumptions rather than unsupported precision.

### 3.2. Reproducibility Across Student Teams Using Standardized Prompts

When executed by student teams using the same prompt sequence, outputs were directionally consistent with the faculty reference run. Teams converged on similar categories of official data sources (government/public health dashboards or administrative reporting portals), demonstrated consistent recognition that identifying procedures requires coding frameworks (and that code sets must be verified within the relevant dataset), and generated comparable interpretive conclusions about local burden and downstream prevention relevance. Variability across teams was largely linguistic and presentational rather than conceptual.

### 3.3. Coding Logic as a Functional Learning and Feasibility Element

Prompts explicitly addressing coding logic elicited consistent differentiation between diagnosis and procedure code systems and highlighted verification steps (e.g., locating dataset documentation, confirming whether the data used ICD-10-PCS, CPT, or diagnosis groupings).

This coding-focused step functioned as a critical bridge between clinical language and public/administrative data structures and reduced the likelihood of teams accepting unverified AI-generated specificity.

### 3.4. Translation from Population Estimates to Pharmacist-Relevant Implications

Across faculty and student executions, the workflow supported translation from population-level estimates into pharmacist-relevant implications, including anticipated encounter frequency (by setting) and prevention-oriented counseling responsibilities. The most consistent practice-level linkage is related to vaccination counseling and prevention strategies relevant to post-splenectomy risk.

### 3.5. Prototype Interface Translation Feasibility

Applying the structured prototype specification prompt (Box 1) generated a multi-screen interface concept that visually mirrored the reasoning structure of SACPH (Figure 1 and Figure 2): context selection, official sources, coding logic, burden estimation with uncertainty framing, clinical risk context, and pharmacist action. The prototype served as a feasibility demonstrator that the text-based workflow can be externalized into an interface-level artifact for broader dissemination and reuse.

Box 1 **Copy-paste prompt to generate a SACPH-style interface concept (prototype specification).**

“You are designing a high-fidelity educational interface concept (not a live app) for health professions students. The purpose is to teach community-anchored data literacy and population-to-practice reasoning.

Use case: [Insert procedure/condition] (e.g., splenectomy/OPSI).

Geography: [Insert county or comparable region].

Time window: [Insert years].

Design requirements:

1. Screen 1: ‘Select Context’ (geography, timeframe, condition/procedure).
2. Screen 2: ‘Official Data Sources’ (show 3–6 official sources with provenance cues and a verification checklist).
3. Screen 3: ‘Coding Logic’ (explain diagnosis vs. procedure codes; how to verify code sets used in the chosen dataset; show uncertainty warnings).
4. Screen 4: ‘Estimated Local Burden’ (display estimates with assumptions; avoid false precision; show a confidence/uncertainty narrative).
5. Screen 5: ‘Clinical Risk Context’ (summarize benchmark risk ranges from literature; avoid patient-specific prediction).
6. Screen 6: ‘Pharmacist Action’ (translate local burden into practice implications: prevention, counseling, anticipated settings).

Constraints:

- Educational demonstration only; no patient-specific advice; no real-time clinical decision support.
- If exact counts cannot be verified, present ranges and describe how the user would verify.

Output format: Provide (a) a wireframe-style description of each screen, (b) the text that appears on each screen, and (c) a short note explaining how this interface reinforces adult learning (the ‘why’) and supports progression on Miller’s Pyramid.”

## 4. Discussion

This work describes the development and feasibility testing of SACPH, an AI-guided inquiry method designed to connect foundational biomedical science instruction with local population health context and pharmacist-relevant reasoning. Rather than evaluating learner outcomes, this study focuses on methodological feasibility and the central question of whether a structured, faculty-authored prompt sequence can reliably support population-to-practice reasoning, and whether that reasoning structure can be translated into a standardized, interface-level artifact suitable for broader dissemination.

### 4.1. Phase 1 Feasibility: Reproducible AI-Guided Population Reasoning

The results of Phase 1 demonstrate that a disciplined, standardized prompt sequence can constrain generative AI behavior in a manner that supports coherent and practice-relevant reasoning.

When executed by faculty and subsequently by student teams using identical prompts (Table 1), AI outputs consistently followed the intended reasoning arc: (1) identification of official public data sources, (2) explicit discussion of coding frameworks used to represent clinical procedures, (3) cautious framing of population-level estimates with stated assumptions, and (4) translation of those estimates into pharmacist responsibilities such as prevention and counseling.

Importantly, feasibility in this study was defined by conceptual convergence rather than numerical precision. Across teams, outputs converged on (1) similar categories of data sources, (2) similar recognition of the distinction between diagnosis and procedure codes, and, importantly, (3) similar interpretations of local burden and downstream implications, even when phrasing differed [16,17,18]. This pattern supports SACPH as a reproducible instructional method rather than a one-off demonstration dependent on individual prompting skill.

### 4.2. Coding Logic as a Critical Educational Bridge

A central contribution of the SACPH workflow is the explicit inclusion of coding logic as part of the inquiry process. Prompts requiring learners to distinguish diagnosis codes from procedure codes and to verify how public datasets operationalize clinical events consistently elicited discussion of data structure, limitations, and uncertainty. This step proved essential for preventing false certainty and for modeling authentic population health reasoning practices.

By surfacing the mechanics through which clinical phenomena are represented in administrative and public health data, SACPH exposes a layer of epidemiologic reasoning that is typically invisible in foundational science instruction. The results suggest that generative AI, when properly constrained, can function as a translator between clinical language and population data structures, supporting data literacy without replacing human judgment.

### 4.3. Phase 2 Feasibility: Translation into an App-Style Interface

Phase 2 of the feasibility study examined whether the validated inquiry logic from Phase 1 could be externalized into a structured, app-style interface.

The figures presented in this manuscript document representative screenshots of a prototype interface designed to mirror the SACPH reasoning sequence. Each figure corresponds to a discrete stage of the workflow defined in the Methods, including contextual specification, presentation of official data sources with provenance cues, explication of coding logic, framing of local burden with uncertainty, and translation to pharmacist-relevant actions.

The significance of these figures lies not in visual design or software completeness, but in demonstrating conceptual portability (Figure 1 and Figure 2). The prototype app shows that the SACPH workflow can be modularized, sequenced, and stabilized beyond text-based interaction with generative AI. This constitutes a second form of feasibility: evidence that population-to-practice reasoning supported by AI can be embedded into an interface-level artifact suitable for repeated use and potential scaling, in other words, a modern-day app. Our app is only for demonstration and uses mock data; further development with live data is lined up for the near future.

### 4.4. Why Interface Translation Matters

The interface translation illustrated in Phase 2 moves SACPH beyond an ad hoc classroom activity toward an infrastructure-ready (app-ready) educational logic. By externalizing the inquiry steps into a visual, sequential format, the prototype reduces reliance on individual prompt craftsmanship and supports standardization across learners and settings. Importantly, the prototype remains intentionally constrained: it does not access live data, does not provide patient-specific recommendations, and is not positioned as a clinical decision tool. These constraints preserve the educational intent of SACPH while illustrating how AI-guided reasoning can be operationalized responsibly. In the future, we plan to extend this into a possibly working app.

### 4.5. Integration of Foundational Science, Population Burden, and Practice Relevance

Taken together, the results from both phases demonstrate that SACPH enables explicit integration of foundational science concepts with community-specific disease burden and pharmacist responsibilities. Although immunology served as the initial use case, the workflow is disease-agnostic and geography-agnostic. The standardized prompt sequence and the interface-level translation both suggest that the method could be adapted to other conditions, regions, and health professions without altering the core reasoning structure.

This integration addresses a persistent gap in health professions education, in which foundational sciences are often taught without reference to local prevalence or anticipated practice encounters. By making population health context visible and locally grounded, SACPH aligns with adult learning principles emphasizing relevance and purpose, while remaining compatible with established models of clinical reasoning development.

### 4.6. AI Literacy and Prompt Engineering as Emerging Professional Competencies

An important implication of this work is that SACPH implicitly teaches AI literacy and prompt engineering as part of professional reasoning rather than as isolated technical skills.

The results demonstrate that learners were required to iteratively engage with AI outputs by refining questions, evaluating explanations, and recognizing the limits of population-level data. This process mirrors emerging expectations that healthcare professionals will need to interact critically with AI systems, understanding not only what outputs are generated, but how those outputs depend on question framing, data provenance, and underlying assumptions [19]. By embedding prompt construction and verification within the progression from “knows” to “shows how”, as depicted in Table 1, SACPH situates AI literacy within established models of clinical competence rather than treating it as a separate or optional skillset. This integration suggests a pathway for teaching responsible AI use that reinforces, rather than disrupts, existing frameworks for professional education.

Although demonstrated in a pharmacy context, the SACPH workflow is readily extensible to interprofessional education by supporting shared interpretation of local population health data within collaborative care teams. Anchoring foundational science concepts to a common data context may facilitate cross-disciplinary reasoning while preserving profession-specific roles [10].

## 5. Limitations and Future Directions

This study is intentionally limited to feasibility and method demonstration. It does not assess learning outcomes, retention, or changes in professional behavior. The workflow relies on the availability and quality of public datasets and requires faculty oversight to verify AI-generated explanations and contextualize uncertainty. The prototype interface is operational with mock data as proof-of-concept.

Future work may formally evaluate educational impact, extend the workflow to additional disease domains and practice settings, and explore integration into experiential and interprofessional education. The prompt-based inquiry instrument and interface specification described here provide a foundation for such extensions while preserving the core principle that AI should support disciplined inquiry rather than supplant professional judgment.

## 6. Conclusions

SACPH demonstrates a feasible, reproducible method for using generative AI to make population health local, visible, and professionally meaningful within pharmacy education. By combining structured prompts with interface-level translation, this work reframes AI as an educational infrastructure capable of supporting community-anchored, data-literate healthcare training rather than a convenience tool for content generation.

## Figures and Tables

**Table 1:** Operationalization of Learning Theory and AI Literacy Within the SACPH Workflow.

Framework	Key Tenet/Level	How It Is Embedded in SACPH Methods	AI Literacy/Prompt-Engineering Competency Embedded
Adult learning theory [8]	Relevance/purpose	Workflow begins with community-specific burden and practice context before mechanistic interpretation	Formulating purpose-driven questions (what matters locally, why it matters)
Adult learning theory [8]	Self-directed inquiry	Learners use structured prompts to identify, verify, and interpret public data sources rather than receiving pre-curated answers	Prompting as inquiry: asking targeted questions; iterating when outputs are incomplete
Miller’s Pyramid [12]	Knows	Identification of foundational concepts and official population data sources	Knowing what can be asked of AI; specifying scope (location, timeframe, condition)
Miller’s Pyramid [12]	Knows how	Interpretation of coding frameworks and population-level estimates; explicit uncertainty framing	Prompt refinement and evaluation: requesting justification, checking assumptions, recognizing uncertainty
Miller’s Pyramid [12]	Shows how	Translation of local epidemiologic context into pharmacist-relevant preventive and counseling implications	Applied AI use: converting AI-assisted findings into practice-oriented reasoning while maintaining verification boundaries

## Data Availability

The original contributions presented in this study are included in the article. Further inquiries can be directed to the corresponding author.
